# Pairwise analysis of gene expression for oral squamous cell carcinoma via a large‐scale transcriptome integration

**DOI:** 10.1111/jcmm.70153

**Published:** 2024-10-29

**Authors:** Nan Li, Zunkai Hu, Ning Zhang, Yining Liang, Yating Feng, Wanfu Ding, Lixin Cheng, Yuyan Zheng

**Affiliations:** ^1^ Department of Stomatology Shenzhen People's Hospital (Second Clinical Medical School of Jinan University; First Affiliated Hospital of Southern University of Science and Technology) Shenzhen Guangdong China; ^2^ Department of Critical Care Medicine Shenzhen People's Hospital (Second Clinical Medical School of Jinan University; First Affiliated Hospital of Southern University of Science and Technology) Shenzhen Guangdong China; ^3^ School of Medicine Southern University of Science and Technology Shenzhen Guangdong China; ^4^ Department of Information and Technology Shenzhen People's Hospital Shenzhen Guangdong China

## Abstract

Among all cancers occurring in the head and neck region, oral squamous cell carcinoma (OSCC) is the most common oral malignant tumours characterized by its aggressiveness and metastasis. The development of transcriptomics technology has greatly facilitated the diagnosis of various cancers. However, identifying genetic biomarkers is limited by data from a single batch of OSCC samples, and integrating analysis across different platforms remains a great challenge. In this study, we integrated five OSCC transcriptome datasets using an innovative strategy capable of mitigating batch effect, and extracting information from different datasets based on changes in the relative expression of gene pairs. By leveraging a machine learning method, we developed a prediction model including 27 differential gene pairs (DGPs) to discriminate OSCC from control samples, achieving an area under the receiver operating characteristic curve (AUC) of 0.8987 for the training set. Moreover, the model demonstrated commendable performance in four external validation sets, with AUCs of 0.9926, 0.9688, 0.8052 and 0.8565, respectively. Subsequently, a prognostic model was constructed based on six key gene pairs through univariate and multivariate Cox regression analysis. The AUCs of the model at 1‐year and 3‐year overall survival time prediction were 0.717 and 0.779 in an independent dataset. Our result demonstrates the effectiveness of this new method of integrating data and identifying DGPs. Using DGPs can significantly improve the performance of both diagnostic and prognostic models.

## 1 INTRODUCTION

Oral squamous cell carcinoma (OSCC) is the most severe malignant tumour among oral cancers.^1^ Globally, Oral squamous cell carcinoma (OSCC) patients account for over 90% of all head and neck squamous cell carcinoma patients, and the mortality rate exceeds 50%.^2^, ^3^ OSCC is a multistep tumour that is usually asymptomatic in the early stages. It initially progresses from mild epithelial hyperplasia to dysplasia and eventually develops into in situ carcinoma, which can lead to late‐stage diagnosis, extensive lesions and cancer metastasis.^4^ Therefore, there is an urgent need to identify genetic markers for OSCC early diagnosis and treatment.

In recent years, with the advancement of sequencing technology, a large amount of transcriptomic data has been accumulated, and identification of genetic markers from transcriptome data has become an important approach for diagnosing complex diseases.^5^, ^6^, ^7^, ^8^, ^9^ However, due to the batch effect of data from different cohorts, researchers are limited to effectively integrate and quanlify omics data from different batches and platforms.^10^, ^11^, ^12^, ^13^ Although some previous studies have reported biomarkers associated with the prognosis of OSCC, they have not effectively integrated datasets from different cohorts to further validate their conclusions due to batch effects from platforms, reagents, and other factors.^14^, ^15^, ^16^, ^17^

Previously, we proposed an algorithm called Individualized Pairwise Analysis of Gene Expression (iPAGE).^18^, ^19^, ^20^, ^21^ This algorithm extracts common information from data sets of different sources and utilizes the relative expression changes of gene pairs to retain the most reliable expression information and guarantee model generalization. iPAGE effectively addresses the issue of batch effects in data, allowing for the integration of data from different sequencing platforms and batches.^22^, ^23^

In this study, we utilized iPAGE to integrate nine gene expression datasets across three companies and five platforms, thereby increasing the sample size for model training. We selected an optimal machine learning algorithm for feature selection and model construction based on the relative expression of differential gene pairs, the prediction model was further validated using four external OSCC datasets. Also, six differential gene pairs were selected to construct a prognostic model via univariate and multivariate Cox regression analysis for OSCC patients.

## 2 MATERIALS AND METHODS

### 2.1 Data set establishment

We conducted a systematic search in the Gene Expression Omnibus (GEO)^24^ database for cohorts that met the inclusion criteria for oral squamous cell carcinoma (OSCC). We obtained a total of 821 samples from 9 cohorts for subsequent analysis. The Cohorts of GSE84846, GSE30784, GSE37991, and GSE85446 were used as the discovery set. Within the discovery set, 80% of the data was used as the training set, and the remaining 20% was used for testing. The training set was used to extract biomarkers and further train the diagnostic prediction model, while the test set was used to evaluate the performance of the model. The GSE25099 dataset was used as the evaluation set to compare the performance of models built by different machine learning algorithms. To validate the generalizability of the final model, GSE89923, GSE31056, GSE85195, and GSE23558 were used as independent external validation datasets. In addition, RNA sequencing data (*n* = 546) and corresponding clinical prognosis information of Head and Neck Cancer patients were downloaded from the UCSC Xena platform to construct a prognosis model.

Datasets from GEO were downloaded using the GEOquery package (Version: 2.72.0) in R environment (Version: 3.6.2), while TCGA datasets were obtained using the TCGAbiolinks (Version: 2.32.0) package. All of these expression data was Log2 transformed to stabilize variance and make the distribution more normal‐like.

### 2.2 iPAGE algorithm

**FIGURE 1:**
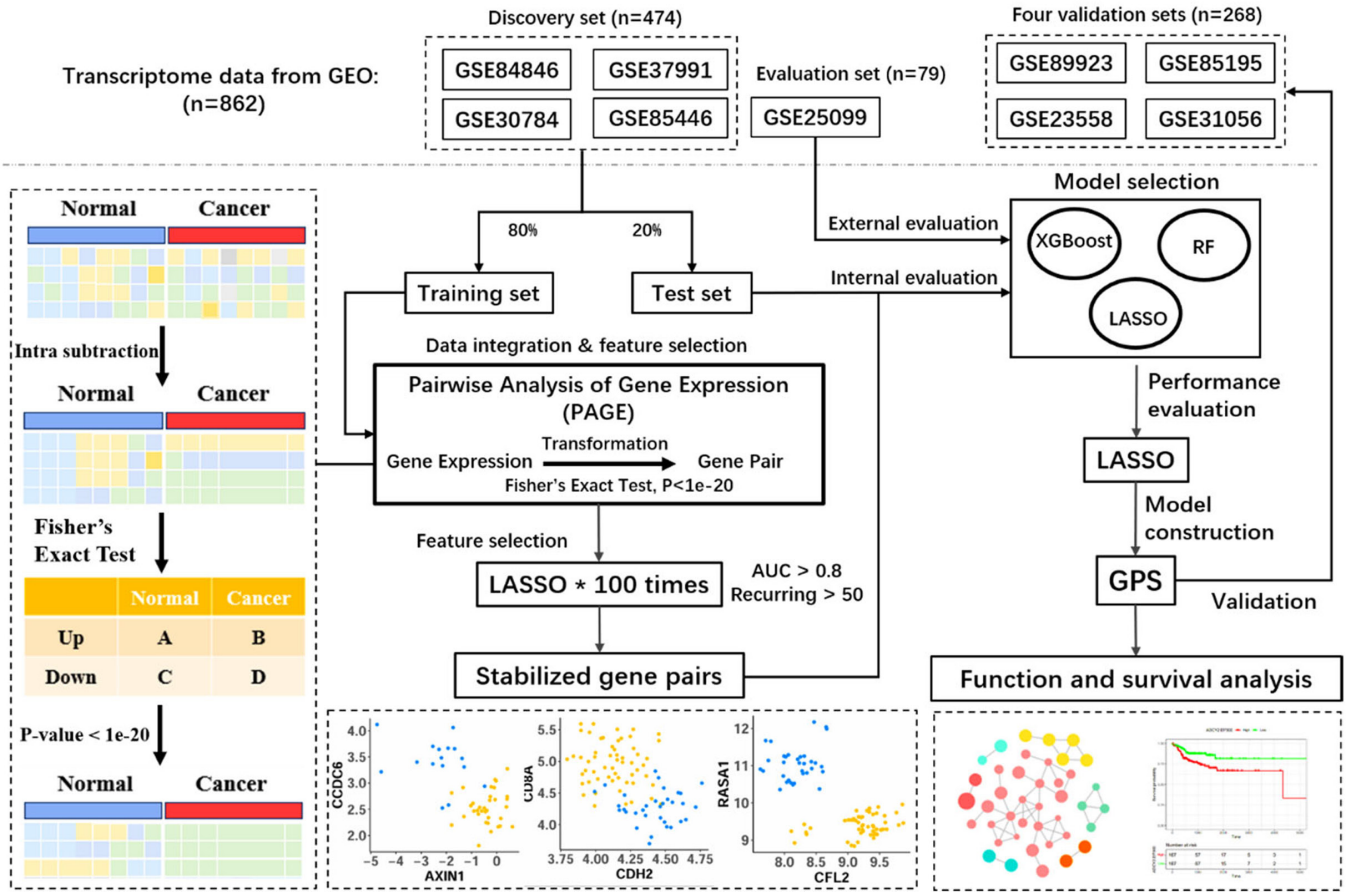
Workflow of this study. A total of 821 gene expression samples across five platforms and three companies were integrated to construct a prediction model for OSCC.

In general, identifying cancer biomarkers requires collecting a sufficient number of sample data. However, the data from different cohorts exhibit batch effects due to various factors, including the use of different amplification reagents, extraction procedures, and sequencing platforms.^25^ Previous studies have shown that the absolute expression abundance of genes is highly influenced by batch effects between cohorts. The genetic markers identified may be inaccurate when integrating cohorts using inappropriate methods.^26^, ^27^, ^28^, ^29^ The iPAGE algorithm is an effective method to integrate data and identified biomarkers.^19^, ^21^ Based on previously published research, we found that the relative expression between specific genes is reversed between the cancer samples and the normal samples. These gene pairs the basis for detecting genetic differences between different cohorts. The relative expression between genes aids in more accurate detection and precise information.^18^, ^20^ Therefore, the relative expression changes between all gene pairs were calculated and using iPAGE to select those gene pairs with stable relative expression changes. The algorithmic workflow of iPAGE is described in Figure 1.

First, we collected 186 gene sets of the Kyoto Encyclopedia of Genes and Genomes (KEGG) pathways from the Molecular Signatures Database (MSigDB) database and gene pairs are extracted within each pathway to capture biologically meaningful genes, as genes involved in the same pathway are typically functionally related. Mathematically, the expression vector for sample k is $\;\left(g_{1}^{\left(k\right)},g_{2}^{\left(k\right)},\ldots ,g_{m}^{\left(k\right)}\right)$. The expression intensity of gene i is $g_{i}^{\left(k\right)}$ for sample k, while label $Y^{\left(k\right)}$ is a binary value with 0 for negative and 1 for positive. The relative expression of gene pair $\left(g_{i}^{\left(k\right)},g_{j}^{\left(k\right)}\right)$ is defined as:

$$r^{\left(k\right)}=I\left(g_{i}^{\left(k\right)}-g_{j}^{\left(k\right)}\right),$$

where $I\left(x\right)=\left\{\begin{array}{c} 1,\text{if}\;x>0\\ -1,\text{if}\;x\leq 0 \end{array}\right.$. If $g_{i}^{\left(k\right)}$ is greater than $g_{j}^{\left(k\right)}$, the relative expression of $\left(g_{i}^{\left(k\right)},g_{j}^{\left(k\right)}\right)$ is 1, otherwise, it is −1.

To quantify the significance of the difference in the relative expression value of a gene pair between the two groups of samples, Fisher's exact test with Bonferroni correction is employed to identify significant gene pairs with reverse expression changes between the cancer group and healthy group.

### 2.3 Feature selection and model construction

The discovery set consists of four cohorts, GSE84846, GSE30784, GSE37991, and GSE85446, which were subsequently split into training and testing sets at a ratio of 8:2, randomly. The training set was used to construct the models, while the testing set was used to validate the model performance. To avoid the random error introduced by the random split of the training and testing sets during feature selection, we performed feature selection 100 times with different random splits of the training and testing sets. To determine the optimal threshold, we set the threshold at intervals of 10 and selected the one perform the best as the optimal parameter. We obtained the highest AUC when the number of iterations reached 50. If the number of iterations is too high, important feature information may be missed, while too few iterations can result in redundant features, leading to model over‐fitting. Gene pairs that appeared in more than 50 selections and with an AUC greater than 0.8 in each selection were considered as candidate biomarkers.

To compare and select the best‐performing model, we employed Random Forest (RF), Least absolute shrinkage and selection operator (LASSO), and eXtreme Gradient Boosting (XGBoost) for model construction.

### 2.4 Model evaluation

To compare the performance of the models, the area under the receiver operating characteristic curve (AUC) was used to assess the models' ability to distinguish positive and negative samples. The higher AUC score indicates better discrimination between the two classes.

### 2.5 Functional enrichment

To explore the functions involved in the identified gene pairs, the hypergeometric distribution was used to enrich gene ontology terms and KEGG pathways. The results were performed using the ‘clusterProfiler’ package in the R environment.^30^

### 2.6 Establishment and evaluation of the prognostic model

We perform univariate regression analysis to identify key genes in OSCC (*p*‐value <0.05) and then subjected to lasso regression analysis using the ‘glmnet’ package in R to construct prognostic model.^31^ The samples are divided into high‐risk and low‐risk groups based on the median risk score. Kaplan–Meier (KM) survival analysis is performed to explore the survival differences between the two groups. The area under the receiver operating characteristic (ROC) curve for 1, 3, and 5 years survival time is calculated using the ‘pROC’ package in R to assess the predictive performance of the model.^32^ To validate the generalizability and reliability of the prognostic model, the GSE31056 dataset is used for validation.

## 3 RESULTS

### 3.1 Data establishment

To train a high‐performance model, a total of 821 samples from nine cohorts were retrieved from the GEO gene expression database. The samples contain 552 OSCC patients and 269 normal controls. The nine cohorts were randomly assigned to three subgroups, namely the discovery set, the evaluation set, and the external validation set (Table 1). In the discovery set (GSE84846, GSE30784, GSE37991 and GSE85446), 80% of the samples (*n* = 379) were randomly selected for model training and 20% (*n* = 95) of the samples were selected for testing. The evaluation set (GSE25099) was utilized to assess the performance of the model. Furthermore, to validate the generalizability of the model, 268 samples from four independent data cohorts (GSE89923, GSE31056, GSE85195 and GSE23558) were used for external validation.

**TABLE 1:** Datasets used in this study.

	Accession Number	OSCC	Normal	Platform
Discovery set (*n* = 474)	GSE84846	99	0	Agilent‐014850 Whole Human Genome Microarray 4x44K G4112F
	GSE30784	167	62	[HG‐U133_Plus_2] Affymetrix Human Genome U133 Plus 2.0 Array
	GSE37991	40	40	Illumina HumanRef‐8 v3.0 expression beadchip
	GSE85446	66	0	Agilent‐014850 Whole Human Genome Microarray 4x44K G4112F
Evaluation set (*n* = 79)	GSE25099	57	22	[HuEx‐1_0‐st] Affymetrix Human Exon 1.0 ST Array [transcript (gene) version]
Validation set (*n* = 268)	GSE89923	57	33	[HG‐U133_Plus_2] Affymetrix Human Genome U133 Plus 2.0 Array
	GSE31056	23	73	[HG‐U133_Plus_2] Affymetrix Human Genome U133 Plus 2.0 Array
	GSE85195	16	34	Agilent‐014850 Whole Human Genome Microarray 4x44K G4112F
	GSE23558	27	5	Agilent‐014850 Whole Human Genome Microarray 4x44K G4112F
Prognosis set(*n* = 546)	HNSC	546	0	TCGA

To perform large‐scale host transcriptome analysis, iPAGE was employed to integrate samples from multiple cohorts. iPAGE is a sophisticated strategy that eliminates batch effects and extracts shared information from different cohorts based on the relative expression changes of gene pairs. Two genes may express fluctuated, but their relative expression within the same individual remains robust against technical differences and batch effects across different cohorts. Hence, we hypothesized that those gene pairs exhibiting consistent relative expression changes between the two groups are most likely to demonstrate expression alterations. Using iPAGE, we integrated all samples from the training set and selected gene pairs with significantly reversed relative expression between the positive and negative groups to construct the prediction model. Since normalization and other numerical transformations have minimal impact on the relative expression of genes, these cohorts were not pre‐processed and only raw data were utilized.

### 3.2 Identification of differential gene pairs

**FIGURE 2:**
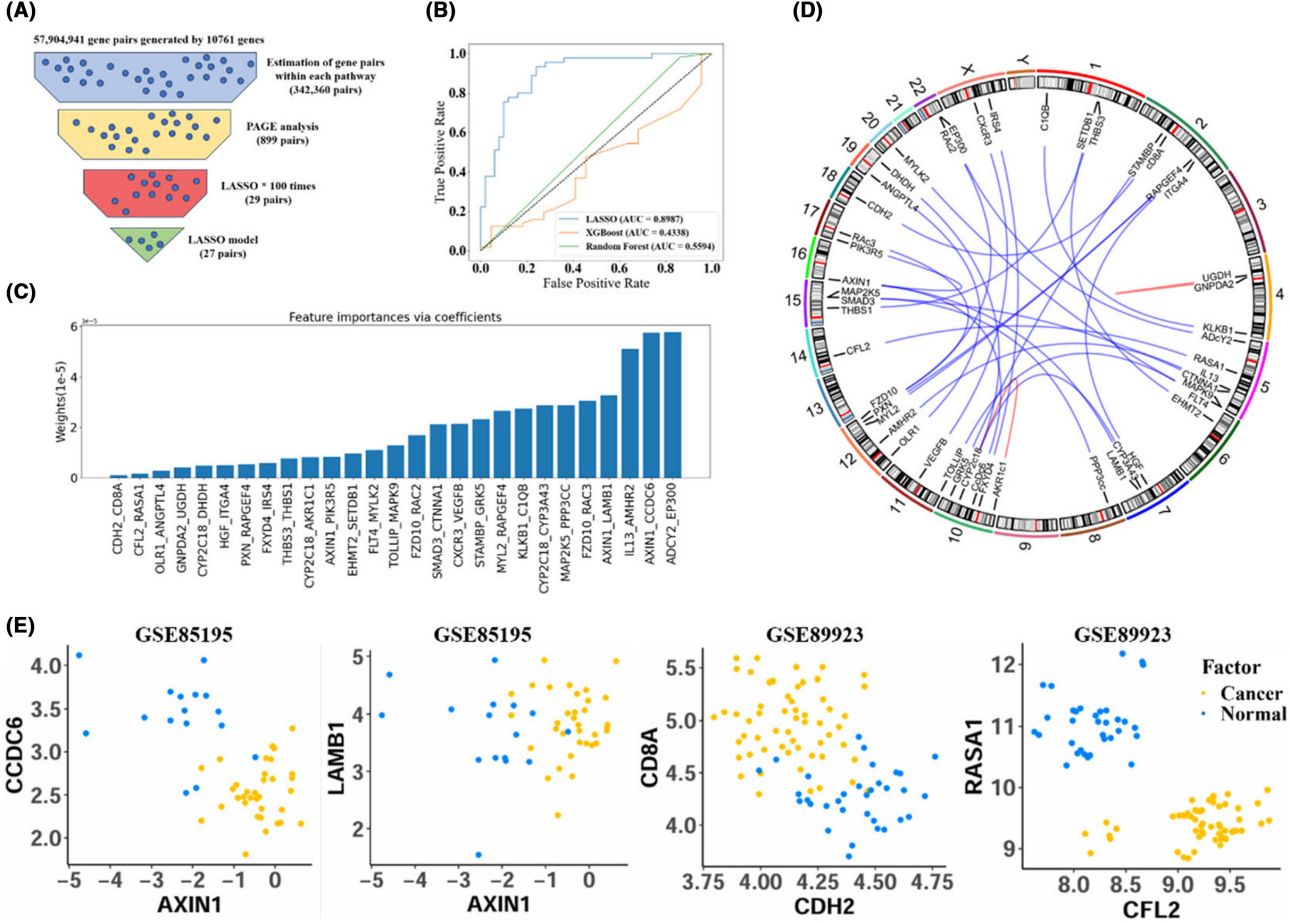
Identification of differential gene pairs. (A) Overview of the screening situation of gene pairs. (B) The translation of the sentence is: The performance of Lasso, XGBoost, and Random Forest on the evaluation set. (C) The weights of the features obtained by the Lasso model. (D) The location and connection of genes on chromosomes, edges linking a pair of genes located on the same chromosome were coloured in red for clear representation. (E) Discriminative ability of gene pairs between OSCC and normal data sets.

A total of 10,762 genes were commonly detected in all datasets. Directly converting these genes into gene pairs would result in over 57 million possible combinations, leading to significant waste of resources and time. The occurrence of cancer is accompanied with changes in the expression levels of genes involved in specific biological pathways. Therefore, we collected 186 KEGG gene sets from the Curated Gene Set in MSigDB.^33^ For each biological pathway, we extracted corresponding genes and constructed gene pairs using the iPAGE algorithm. Subsequently, Fisher's Exact Test was employed to identify DGPs with the largest reverse expression differences between the OSCC and control groups. Bonferroni correction was applied to control the false discovery rate at a significance level of 10^−20^, resulting in 899 DGPs ultimately (Figure 2A).

### 3.3 Model construction and validation

80% of the discovery samples was randomly assigned as the training set for feature selection using LASSO. LASSO penalizes redundant and correlated gene pairs to select the minimum number of gene pairs. The remaining 20% of the data was used as the test set to evaluate the performance of LASSO in penalizing redundant genes. This procedure was repeated 100 times to ensure that the selected gene pairs are not influenced by the randomness of the dataset split. The gene pair with an AUC greater than 0.8 and appeared in at least 50 out of 100 iterations were selected, ultimately resulting in 29 gene pairs.

To compare the predictive performance of three machine learning algorithms, RF, XGBoost, and LASSO, we calculated the AUC of the three models in an evaluation set (GSE25099), achieving AUCs of 0.6124, 0.4338 and 0.8987, respectively (Figure 2B). LASSO was the optimal one and thus was used to build the prediction model. It was determined the optimal parameter alpha = 0.89 using ten‐fold cross‐validation. Finally, we obtained 27 DGPs that can effectively distinguish between the OSCC and the control samples using LASSO (Figure 2C). The genome locations of these genes and their connection are shown in Figure 2D. As shown in the PAGE plots, a single gene cannot sufficiently discriminate OSCC from the control samples, but a gene pair separates the two groups more effectively (Figure 2E).

**FIGURE 3:**
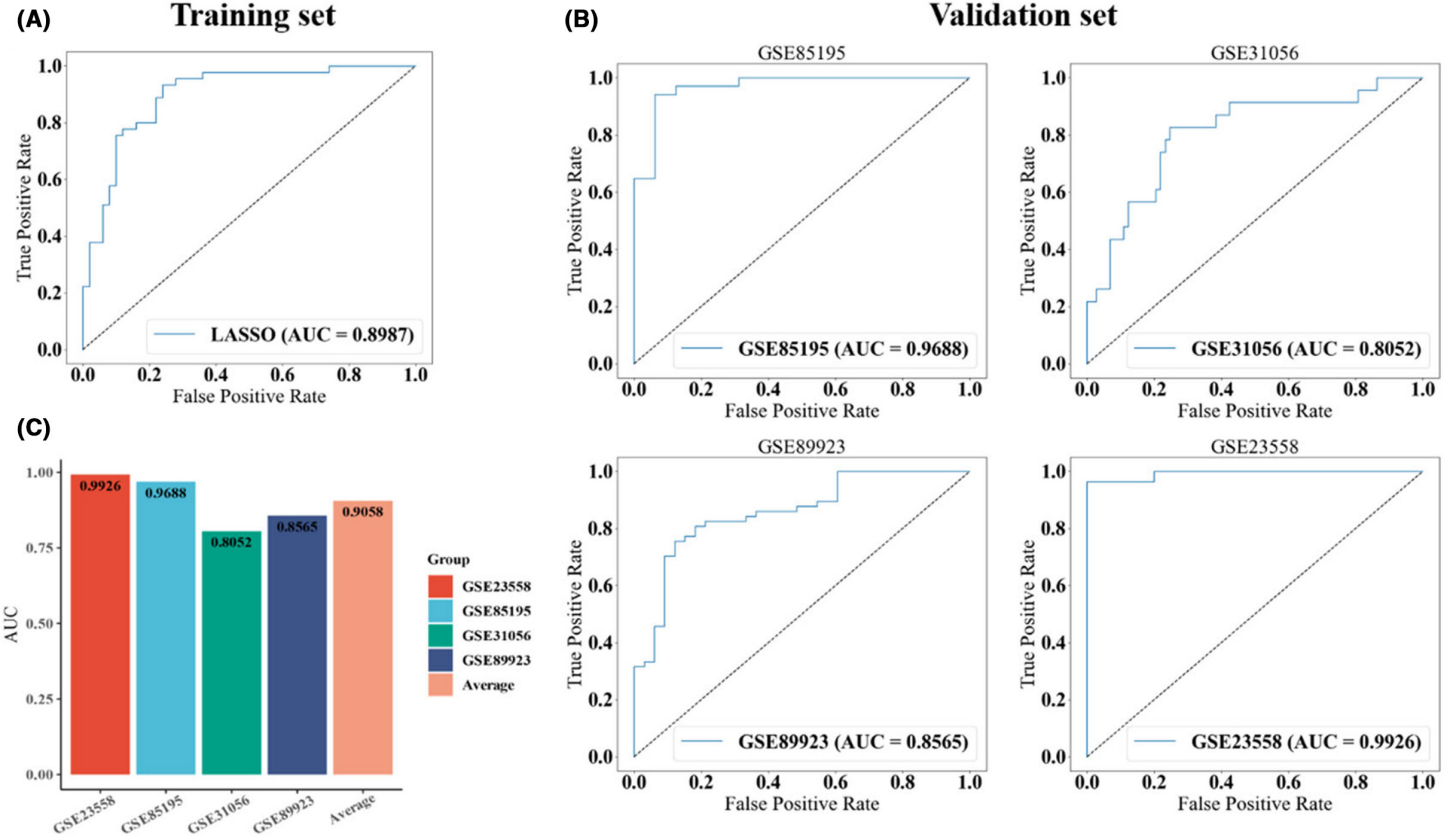
Performance evaluation. (A) ROC curve of the training set. (B) ROC curves of four validation set. (C) Average AUC score of the four validation sets.

To evaluate the generalization of the model in different platforms, four external cohorts from Gene Expression Omnibus (GEO) were selected for independent validation, including GSE89923, GSE31056, GSE85195, and GSE23558. The AUCs of our model in the four cohorts were 0.9926, 0.8052, 0.9688, and 0.8565, respectively (Figure 3A,B). Overall, the average AUC across the four datasets was 0.9058, demonstrating the robustness of this model across two gene expression detection platforms, i.e., Affymetrix (HG‐U133_Plus_2) and Agilent (4x44K G4112F). These results reveal the potential of iPAGE as a reliable tool for identifying OSCC biomarkers, providing insights for the early detection and personalized treatment of OSCC (Figure 3C).

### 3.4 Functional characterization of the differential gene pairs

**FIGURE 4:**
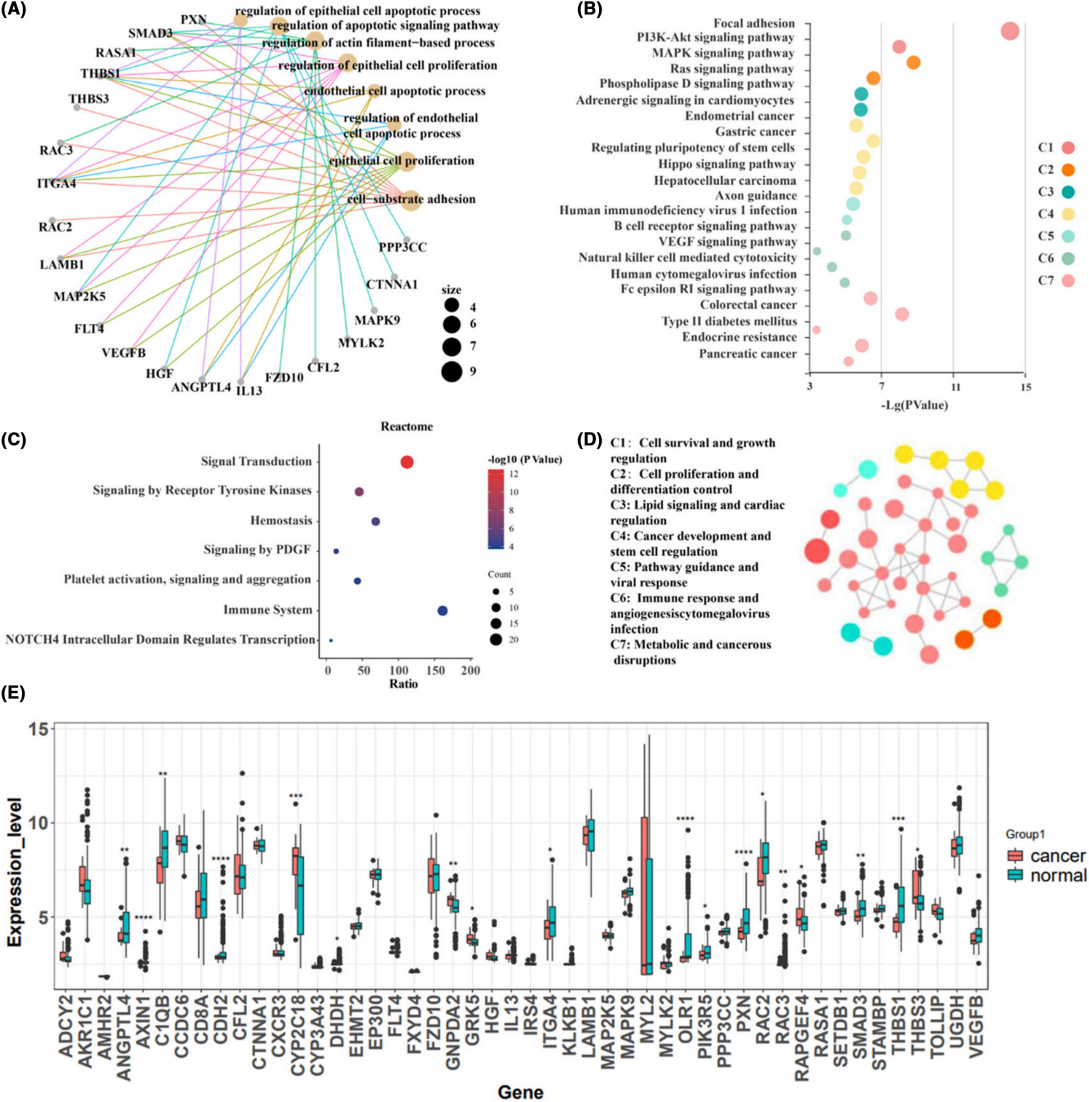
Function characterization of differential gene pairs. Functional enrichment analysis of gene pairs in GO (A), KEGG (B), and Reactome (C). (D) Functional clustering in KEGG; (E) The expression levels of genes in gene pairs in the validation set.

We further explored the biological functions and pathways involved in the DGPs of the model. Gene Ontology functional analysis revealed that these gene pairs are mainly involved in biological processes such as epithelial cell proliferation, cell proliferation, and apoptosis, which are closely related to cancer (Figure 4A). KEGG analysis also showed that these genes are involved in cancer related pathways such as the VEGF signalling pathway and MAPK signalling pathway (Figure 4B,D). Vascular endothelial growth factor (VEGF) is the most prominent protein among the angiogenic cytokines and is believed to play a central role in neoangiogenesis in cancer and other inflammatory diseases.^34^ The MAPK pathway is dysregulated in many RAS‐associated cancers.^35^ Similarly, enrichment analysis using the Reactome database revealed these genes are also related to immunity and cancer pathways, and BOTCH4 is found to be associated with tumour occurrence (Figure 4C). For most of the genes in the DGPs, the expression distributions between cancer and normal groups are not significantly different in the validation set (Figure 4E), whereas the constructed DGPs are powerful in discriminating cancer samples from the normal ones.

### 3.5 Prognostic value of differential gene pairs

**FIGURE 5:**
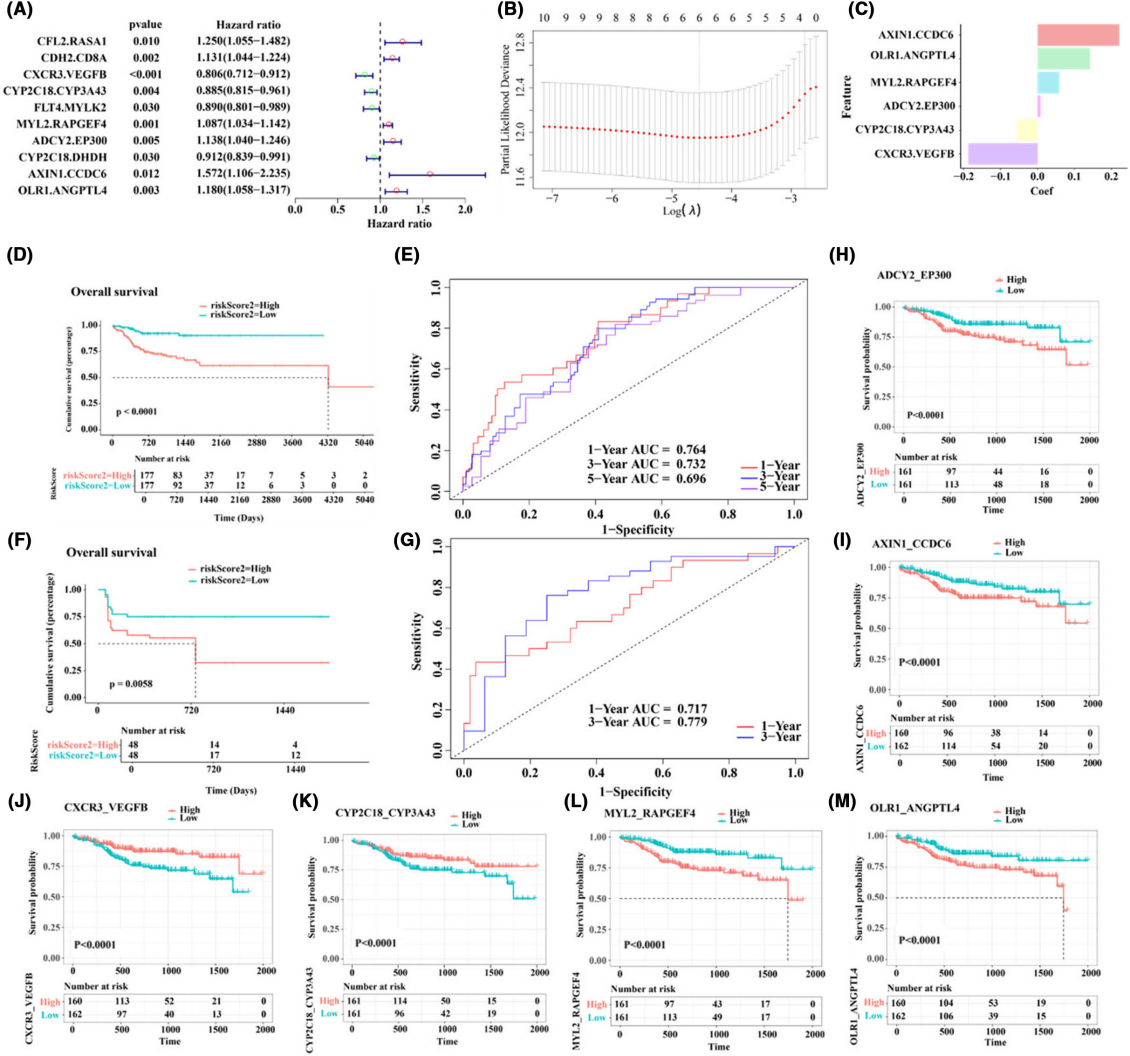
Prognosis evaluation of the differential gene pairs. (A) Univariate Cox regression forest plot; (B, C) Multivariate Lasso‐Cox regression feature weights; Kaplan–Meier analysis of the training set (D) and of the validation set (F); AUC curves of 1, 3, and 5‐year survival models for the training set (E) and for the validation set (G);(H–M)Kaplan–Meier analysis of the GPS.

To explore the relationship between identified DGPs and the clinical prognosis of patients, univariate Cox analysis was performed on the 27 DGPs, and 10 DGPs were identified as potential prognostic molecular markers (Figure 5A,B). DGPs associated with the favourable prognosis are highlighted in green, while genes associated with poor prognosis are highlighted in blue. LASSO‐Cox regression was used to select DGPs that were significantly associated with OSCC prognosis, resulting in the retention of six DGPs with non‐zero coefficients (Figure 5C). A multivariable prognostic model was established based on these DGPs to predict the survival time of OSCC patients (Figure 5D,F).

To evaluate the prognostic value of the model, time‐dependent ROC curves were used to describe the predictive performance. The prognostic model demonstrated excellent performance in both the training and validation datasets. In the training set, the AUC of the model at 1 year, 3 years, and 5 years OS prediction was 0.764, 0.732, and 0.696, respectively. In the validation set, the AUCs of the model were 0.764 and 0.732 at 1‐year and 3‐year OS prediction, respectively. Due to limited clinical information in the validation dataset (GSE31056), the 5‐year survival time could not be predicted (Figure 5E,G). We then conducted a survival analysis on the six non‐zero coefficient DGPs selected by LASSO‐Cox regression, which were significantly associated with OSCC prognosis. The results indicated a significant difference in survival probability between patients divided into two groups based on the relative expression of the six DGPs (Figure 5H–M). Specifically, patients in the low‐expression groups of MYL2_RAPGEF4, OLR1_ANGPTL4, AXIN_CCDC6, and ADCY2_EP300 had higher survival rates compared to those in the high‐expression groups. Conversely, for CYP2C18_CYP3A43 and CXCR3_VEGFB, patients in the high‐expression groups exhibited higher survival probabilities.

Gene Ontology enrichment analysis was performed on the OSCC prognosis‐related genes (Figure S1). These genes are involved in functions of ‘vascular endothelial growth factor receptor 1 binding’, which is critical for tumour angiogenesis. Pathways involved in steroid metabolism, such as ‘testosterone 6‐beta‐hydroxylase activity’ and ‘steroid hydroxylase activity’, are also significantly enriched, suggesting a role in regulating the tumour microenvironment. Furthermore, ‘oxidoreductase activity’ highlights the importance of redox balance in OSCC cells, potentially affecting cancer progression and patient prognosis.

## 4 DISCUSSION

Using the iPAGE algorithm, we carried out a large‐scale integrative analysis of ten OSCC transcriptome datasets among three companies and five platforms. We identified 27 differential gene pairs as biomarkers for OSCC using LASSO. Through univariate and LASSO‐Cox regression analysis, we further selected prognostic biomarkers associated with OSCC, resulting in a total of six prognostic‐related gene pairs. Through validating in an independent dataset, we demonstrated the significance of these biomarkers for OSCC prognosis.

Due to the batch effects of datasets from different platforms, machine learning models were often trained and validated on a single dataset,^36^, ^37^, ^38^ which can potentially reduce the accuracy and robustness of the models. iPAGE enhances the power of feature selection by integrating multiple datasets among different resources, which can increase the scale of data and improve the performance of the model.

In addition, we identified six DGPs significantly associated with the survival of OSCC and built a risk model for prognosis prediction. Among the genes in this model, some have been reported in previous research. For instance, the expression of ADCY2 is related to open chromatin regions in radioresistant OSCC cells, and ADCY2 might have therapeutic effects when combining with radiotherapy in OSCC patients.^39^ XIST enhances the growth and invasion of OSCC cells by targeting the miR‐133a/VEGFB axis (K.^40^). CXCL11 may affect the expression of CD274 and IDO1 in an autocrine approach in OSCC.^41^ These findings demonstrated the potential of these gene pairs to serve as targeted biomarkers for OSCC treatment.

The DGPs identified in the current study that are associated with OSCC prognosis require further clinical and experimental validation. Increasing the sample size in the study population, and conducting more comprehensive and detailed follow‐up are needed to further confirm the findings. In future work, once sufficient data is available, we will promptly collect the latest database to build a larger training set to construct a more accurate model.

## AUTHOR CONTRIBUTIONS

**Nan Li:** Data curation (equal); writing – original draft (equal); writing – review and editing (equal). **Zunkai Hu:** Data curation (equal); formal analysis (equal); visualization (equal); writing – original draft (equal). **Ning Zhang:** Writing – review and editing (equal). **Yining Liang:** Writing – review and editing (equal). **Yating Feng:** Writing – review and editing (equal). **Wanfu Ding:** Methodology (equal); project administration (equal); supervision (equal). **Lixin Cheng:** supervision (equal); methodology (equal); writing – review and editing (equal). **Yuyan Zheng:** Funding acquisition (equal); writing – review and editing (equal).

## CONFLICT OF INTEREST STATEMENT

The authors have no conflicts of interest to declare.

## FUNDING INFORMATION

This work was supported by Natural Science Foundation of China (no. 32000516) and Shenzhen Science and Technology Research and Development Fund (JCYJ20190809165805604).

## Supporting information


Figure S1.

## Data Availability

The data underlying this article are available in the GEO database, at https://www.ncbi.nlm.nih.gov/geo/. Data used for training and testing are available in Table 1.
