# Supplementary figures and images for: Pairwise analysis of gene expression for oral squamous cell carcinoma via a large‐scale transcriptome integration
Source: J Cell Mol Med. 2024 Oct 29;28(20):e70153. doi: 10.1111/jcmm.70153 (PMC11520439; doi:10.1111/jcmm.70153)

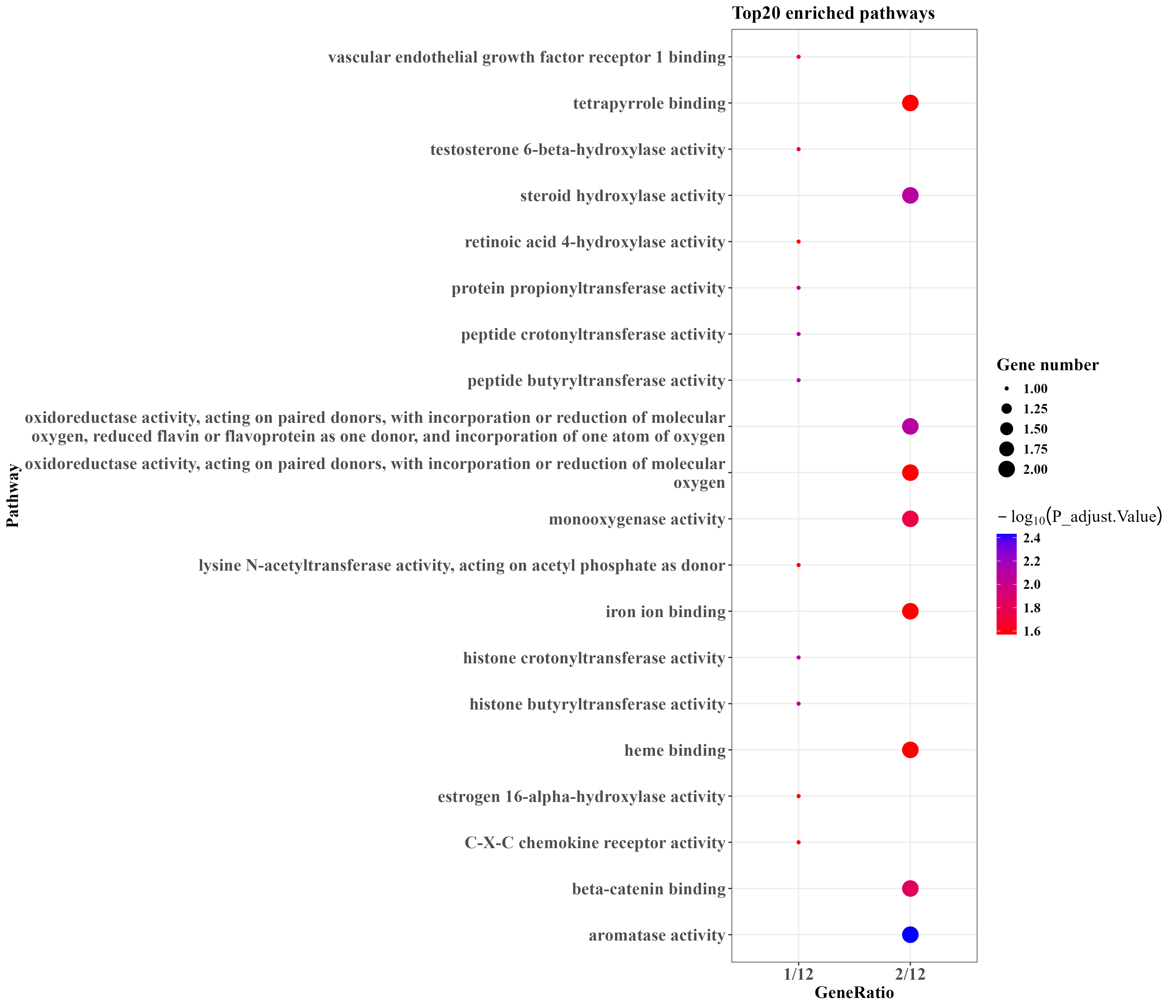

Supplement: Supplementary file 1 — Figure S1. [file JCMM-28-e70153-s001.png]
